# A Clinical Lecture on Goitre

**Published:** 1898-05-28

**Authors:** J. Lacy Firth

**Affiliations:** AssistantSurgeon to the Bristol General Hospital


### Medical Progress and Hospital Clinics.

[*The Editor will be glad to receive offers of co-operation and contributions from members of the profession. All letters should be addressed to* The Editor, at the Office, 28 & 29, Southampton Street, Strand, London, W.C.]

### A CLINICAL LECTURE ON GOITRE.

By J. Lacy Firth, M.D., M.S., F.R.C.S., Assistant-Surgeon to the Bristol General Hospital.

I have recently had under my care some cases of chronic enlargement of the thyroid gland, and have therefore selected that subject for consideration to-day. I need hardly remind you of the structure of the thyroid gland, of its vesicles lined with a single layer of cubical epithelium and containing a clear albuminous fluid, and of the very vascular areolar tissue binding together and supporting the vesicles. All the chronic general enlargements of the gland begin in the same way as a pure hypertrophy, but often, as the enlargement progresses, one or other of the constituent elements of the gland increases out of proportion to the others. These general enlargements of the gland are termed goitres or bronchoceles. If all the elements of the gland are hypertrophied in proportion the swelling is called a simple or parenchymatous goitre or bronchocele. If the vesicles more especially enlarge a cystic goitre is formed; if the fibrous tissue especially develop, a fibrous goitre; and if the vessels especially increase in size and number, a pulsating goitre results.

Frequently, however, the enlargement is due to the growth of encapsuled tumours within the gland. These tumours are adenomata, and have a structure apparently identical with that of the gland in which they arise. Such an enlarged thyroid is sometimes called an adenomatous goitre. These encapsuled growths vary much in size, but are usually between a cherry and a hen's egg. As they enlarge, often neighbouring vesicles in them coalesce, producing larger and larger spaces, and finally one single cavity with a fibrous wall may alone remain, all trace of the original glandular tissue being lost. By some writers the term bronchocele is applied to these cysts only. They may be very large. One has been described which was so large that it reached nearly to the navel, and was so heavy that the patient was accustomed to rest it upon a table for relief. Some writers prefer not to apply the term goitre to the swellings produced by these encapsuled tumours, whether they be solid, cystic, or mixed solid and cystic, because, in a sense, the whole thyroid gland is not enlarged by them. These writers speak of these swellings as adenomata of the thyroid, if solid, and as cysts of the thyroid if cystic. They discard the term adenomatous goitre, and reserve the name of cystic goitre for those cases in which the cysts are not encapsuled, and are developed by the enlargement and coalescence of the original vesicles of the gland, not from the vesicles of an adenoma. The important practical point to remember is that, as a rule, adenomata, whether cystic or solid, shell out easily, and may therefore be removed without extirpation of any part of the gland itself.

A parenchymatous or adenomatous goitre is sometimes associated with complex nervous, circulatory, and other symptoms, notably proptosis of the eyes, constituting the disease known as exophthalmic goitre.

Rarer forms of enlargement of the thyroid are those due to tuberculous or gummatous disease, or to the presence of hydatids, or to the growth in the gland of a primary sarcoma or carcinoma, or its invasion by epithelioma from the adjacent mucous membrane of the trachea or œsophagus. Malignant disease of the thyroid shows but slight tendency to produce metastatic deposits, but a few very curious and interesting cases, have been recorded in which tumours composed of thyroid tissue have appeared on bones, especially on those of the skull, and in all these cases the thyroid gland was enlarged.

The swelling formed by an enlargement of the thyroid may be symmetrical, and of the same shape as the normal gland. This is the rule in chronic simple goitre. Normally the right lobe of the gland is slightly larger than the left, and in hypertrophy this difference in the size of the two lobes is often exaggerated, as one would naturally expect. When the enlargement is due to the development of adenomata, solid or cystic, it is usually unsymmetrical. Often it is of one side only. There may be symmetrical adenomata, *e.g.*, one in each lobe, but the rule is that chronic unsymmetrical enlargement is caused by the growth of these tumours. Whether the gland be enlarged symmetrically or unsymmetrically, the diagnosis is generally easy, because the swelling is closely attached to the trachea, and moves upwards with it in swallowing. Also, the isthmus can generally be felt passing from the swollen lobe across the front of the trachea. If one lobe is enlarged and pulsates vigorously, it may simulate an aneurism. but usually the pulsation will be much diminished if the lobe be lifted forwards from the carotid, for the pulsation is largely transmitted from that artery by contact. It is often difficult to decide whether the enlargement is solid or cystic, for the solid growth is frequently soft and elastic, yielding a sensation like that of fluid, and, on the other hand, a cyst may be so tense as to feel like a solid growth, and may even have its wall hardened by calcification.

Fibrous goitre is more often read about than met with. A hard, firm goitre is usually parenchymatous, the hardness being due to the tensity of the distended vesicles. I must mention that the movement with deglutition may be lost early in the course of malignant disease.

The symptoms which an enlargement of the thyroid produces are the result either of the pressure it exerts upon the surrounding structures, or of an alteration in the quantity or quality of its so-called "internal secretion." Of these symptoms, that of dyspnœa is the most important. The dyspnœa may be induced by exertion, when the accelerated respiration will be accompanied by loud stridor and more or less lividity. Apart from exertion, however, some persons with enlarged thyroids are subject to sudden attacks of dyspnœa without obvious exciting cause, and rapid or sudden death may result. These attacks often begin during sleep. Often patients are, therefore, afraid to go to sleep, or they may be unable to sleep lying down, though they can if propped up.

(*To be continued.*)

